# Advances in Lignin Chemistry, Bonding Performance, and Formaldehyde Emission Reduction in Lignin‐Based Urea‐Formaldehyde Adhesives: A Review

**DOI:** 10.1002/cssc.202500491

**Published:** 2025-07-12

**Authors:** Anass Ait Benhamou, Liza Abid, Ingrid Calvez, Alain Cloutier, Mojgan Nejad, Tatjana Stevanovic, Véronic Landry

**Affiliations:** ^1^ Department of Wood and Forest Sciences Renewable Materials Research Centre (CRMR) Université Laval Quebec QC G1V 0A6 Canada; ^2^ Department of Forestry Michigan State University 480 Wilson Rd East Lansing 48824 Michigan USA

**Keywords:** composites, formaldehyde, lignin, urea‐formaldehyde adhesives, wood

## Abstract

Lignin, a complex biopolymer derived from plant biomass, has attracted significant attention in both academic research and industry due to its potential to revolutionize formaldehyde‐based adhesives by reducing their emissions, a critical concern in the wood industry. In the realm of wood adhesives, the integration of lignin has seen significant progress in recent years, where it is utilized either as a partial replacement for traditional synthetic resins or as a modifier to enhance adhesive properties. This has led to notable improvements in both environmental impact and adhesive performance, contributing to developing more sustainable and ecofriendly wood composite materials. This review provides an in‐depth exploration of the recent advancements in the rapidly growing field of lignin‐based urea‐formaldehyde (UF) adhesives, spanning from fundamental research to practical applications. The initial sections of this article offer an updated overview of lignin, covering its chemical structure, properties, extraction methods, and various chemical modifications, with a focus on its potential in adhesive applications. The review concludes with a detailed discussion of the economic and environmental advantages, alongside the challenges and future directions for integrating lignin into UF adhesive technology. This review play a key resource for understanding the evolving field of sustainable wood adhesives.

## Graphical abstract

This review presents a comprehensive overview of recent progress in lignin chemistry and its application in urea‐formaldehyde adhesives. Key advances in lignin modification, bonding performance, and strategies for reducing formaldehyde emissions are critically discussed, highlighting pathways toward greener wood adhesive technologies.

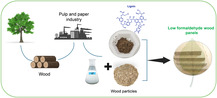

## 1 Introduction

In regions with high population density and intense industrial activity, air pollution is recognized as a primary environmental concern, posing significant risks to public health and the ecosystem.^[^ ^1^ ^]^ Nowadays, discussions about air quality have become more pressing, especially regarding indoor gases, which directly impact human health.^[^ ^2^ ^]^ The major contributors to air pollution are vehicles and industrial activities, which burn fossil fuels. These activities release significant amounts of volatile organic compounds (VOCs), including nitrogen oxides (NOx), sulfur oxides (SOx), and carbon monoxide (CO), into the atmosphere.^[^ ^3^, ^4^ ^]^

Formaldehyde (FA) is one of the most common indoor air pollutants.^[^ ^5^, ^6^ ^]^ It is a colorless gas that can polymerize under certain conditions, though it typically remains in its monomeric form at room temperature.^[^ ^7^ ^]^ This compound is released into the environment from sources such as furniture, flooring materials like plywood, adhesives, paints, and insulation materials.^[^ ^2^, ^5^, ^8^, ^9^, ^10^, ^11^, ^12^, ^13^ ^]^ Given that people spend ≈88% of their time indoors, combined with the prolonged‐release cycle of FA from various furniture and decorative paints, the risks associated with exposure are significant.^[^ ^6^, ^14^ ^]^ The harmful effects of FA were first reported in the mid‐1960s when it was linked to health issues in prefabricated houses, including eye and throat irritation.^[^ ^15^ ^]^

Long‐term and short‐term FA exposure generally enhances the risk for respiratory problems and toxicity.^[^ ^6^, ^16^ ^]^ Moreover, prolonged exposure is also associated with an increased risk of cancer incidence.^[^ ^14^, ^16^, ^17^, ^18^, ^19^ ^]^ About 28 million people worldwide are estimated to be affected annually by indoor FA pollution^[^ ^20^ ^]^ and more consideration needs to be directed towards this issue. Specifically, more effective techniques for mitigating FA emissions should be developed. Based on epidemiological studies, the World Health Organization (WHO) established an indoor air quality guideline in 2010 for short‐ and long‐term exposures to FA of 01 mg m^−^^3^ (0.08 ppm) for all 30‐minute periods over a lifetime.^[^ ^2^, ^21^ ^]^

Adhesives are crucial for efficiently utilizing wood resources and advancing the forest products industry.^[^ ^22^ ^]^ Between 1900 and 1930, FA‐based resins became key adhesives for wood and composites.^[^ ^8^ ^]^ During World War II in Bremen, Germany, particleboards were increasingly recognized and became the most important alternative to solid wood for housing construction by 1950.^[^ ^8^ ^]^ They now account for ≈63% of all wood‐based panel products.^[^ ^23^ ^]^ UF adhesives are commonly used in panel manufacturing, accounting for over 90% of global board production^[^ ^19^, ^24^ ^]^ due to their ease of processing, high reactivity, low cost, strong wood adhesion, and excellent thermal properties.^[^ ^25^, ^26^, ^27^, ^28^, ^29^ ^]^ The main shortcoming of these adhesives is their poor water resistance, along with the FA emission, which is carcinogenic.^[^ ^27^ ^]^

Over the past decade, several studies have explored various methods to reduce FA emissions and improve adhesive properties in wet conditions. These approaches include incorporating FA scavengers such as glycidyl ether, melamine, phenol, and acrylamide^[^ ^24^, ^30^ ^]^ as well as using natural biopolymers like protein, soy, starch, chitosan, and gums as partial replacements of adhesive formulations.^[^ ^27^, ^31^, ^32^, ^33^ ^]^ Lignins are polymers of great interest in the development of bio‐based wood adhesives, particularly phenol‐formaldehyde (PF) resins.^[^ ^18^, ^34^, ^35^, ^36^, ^37^, ^38^ ^]^ In biomass, lignin acts as a natural matrix that maintains the structure of cellulosic fibers, contributing to the plant's rigidity and strength, facilitating water transport, and offering protection against environmental damage.^[^ ^12^ ^]^ Indeed, lignin's ability to bind with FA molecules can diminish emissions, creating more environmentally friendly wood products.^[^ ^39^, ^40^ ^]^

**Figure 1:**
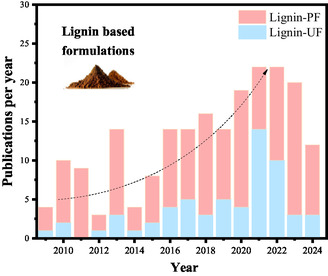
Annual number of scientific publications in the last decade using the following data.

Despite the growing body of research on various adhesive technologies, the incorporation of lignin into UF adhesives remains relatively underexplored. In recent years, there has been an increasing interest in lignin as a potential phenol replacement due to its abundance and unique chemical properties, such as its high aromatic content, complex polyphenolic structure, and potential for chemical modification, which makes it suitable for enhancing the performance of bio‐based materials. However, compared to the extensive research and numerous patents related to PF adhesives, lignin's application in UF adhesives has not received the same level of attention (**Figure** **1**). This gap highlights a significant opportunity for innovation. Exploring lignin's role in UF adhesives could lead to advancements that enhance adhesive performance and contribute to more sustainable and environmentally friendly solutions. The gradual rise in publications over the past decade underscores a burgeoning interest, suggesting that further research could unlock lignin's full potential as a valuable component in UF adhesive formulations.

This review provides an in‐depth analysis of the use of lignins in wood‐based adhesives, encompassing various forms such as Kraft lignin and chemically modified lignin. We begin by examining the fundamental properties of lignin, including its chemistry and extraction processes. The review also delves into various chemical modifications of lignin and their potential applications in improving the performance of wood adhesives. Additionally, we provide an overview of the latest research on lignin's role in UF adhesives for wood bonding. The review concludes with an analysis of the economic and environmental benefits, as well as the challenges and future directions. This comprehensive examination aims to shed light on “lignins” potential to improve UF adhesive formulations and to guide future research in this promising area.

## 2 Lignin Fundamentals

### 2.1 Chemistry of Lignin

**Figure 2:**
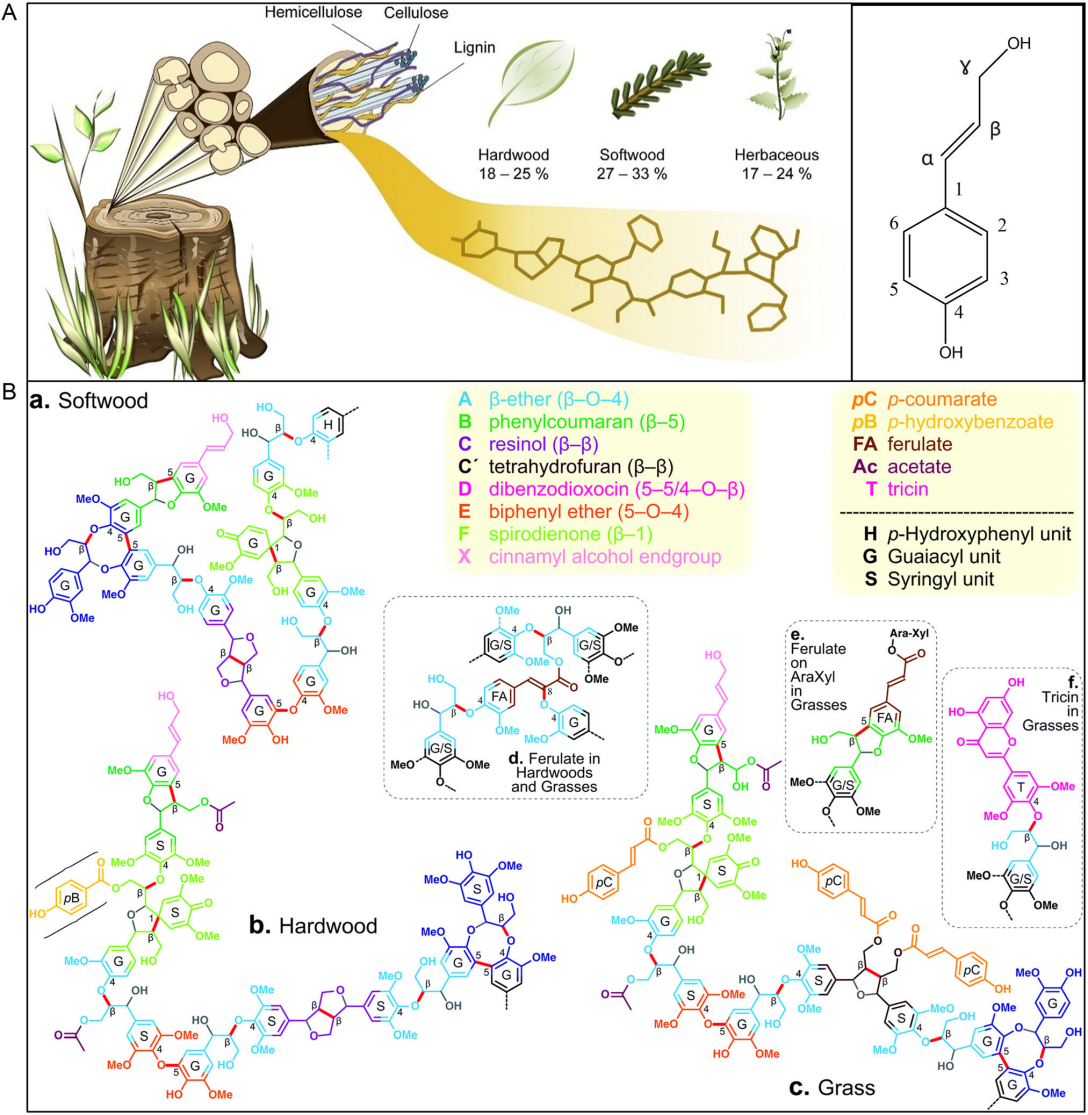
a) Structural organization and major components in plant cell walls, as well as lignin contents in different sources. Reproduced with permission.^[^ ^42^ ^]^ Copyright 2019, Elsevier. Representation of lignin structures with 11 units for: (a) Softwood, b) hardwood, and c) grass, including the major chemical linkages. Reproduced with permission.^[^ ^128^ ^]^ Copyright 2021, Royal Society of Chemistry.

Lignin is one of the major components of the lignocellulosic cell wall, alongside cellulose and hemicellulose. Quantitatively, it is the second most abundant biopolymer on Earth after cellulose.^[^ ^41^ ^]^ It constitutes about 15% to 25% of hardwood, 25% to 35% of softwood, and 15% to 25% of herbaceous plants (**Figure** **2**).^[^ ^42^ ^]^ Lignin is a hyperbranched biopolymer composed of phenylpropane units. Its heterogeneous structure results from the oxidative coupling of three monolignols derived from cinnamic alcohol: *p*‐coumaryl alcohol, coniferyl alcohol, and sinapyl alcohol. The heterogeneity of lignins stems from the variable proportions of these three units and the diversity of linkages between them.^[^ ^43^ ^]^ This variability depends on the species and plant tissue from which they originate. The basic monomer of lignin consists of an aromatic ring with an attached side chain linked at the C‐1 position. The oxygen of the phenol group is fixed at the C‐4 position of the aromatic ring.^[^ ^44^ ^]^ The carbon atoms of the side chain are designated by the letters *α*, *β*, and *γ*, indicating their respective positions along the chain, as depicted in Figure 2.^[^ ^44^ ^]^

The phenylpropanoid units, namely hydroxyphenyl (H), guaiacyl (G), and syringyl (S), are linked by carbon–carbon (C—C) and ether (C—*O*—C) bonds. Among these, the *β*—*O*—4 ether bond is the most abundant in lignins, regardless of their source, constituting 50% to 80% of the linkages (Figure 2)^[^ ^45^, ^46^ ^]^ Other types of linkages also connect the subunits, such as *α*‐*O*‐4, *β*‐5, *β‐β*, and 5‐5, which vary significantly depending on the type of lignin.^[^ ^42^ ^]^ Based on the relative quantities of H, G, and S units, three types of lignins are distinguished: softwood lignin rich in guaiacyl, often referred to as G lignin; hardwood lignin characterized by an almost equal proportion of guaiacyl and syringyl units, known as GS lignin; and finally, lignin from grasses and herbaceous plants containing all three units, classified as G/S/H type.^[^ ^47^ ^]^

Lignins are composed of various functional groups, such as methoxy (CH_3_ *O*‐), carboxyl (‐COOH), carbonyl (C=O), and phenolic and aliphatic hydroxyl groups. These functional groups give lignins high chemical reactivity, thus expanding its potential applications. Depending on its structure, which is directly linked to the lignin isolation process, it can be used to design adhesives, coatings, bioplastics, fuel, etc.^[^ ^47^ ^]^

### 2.2 Lignin Types and Extraction Processes

**Figure 3:**
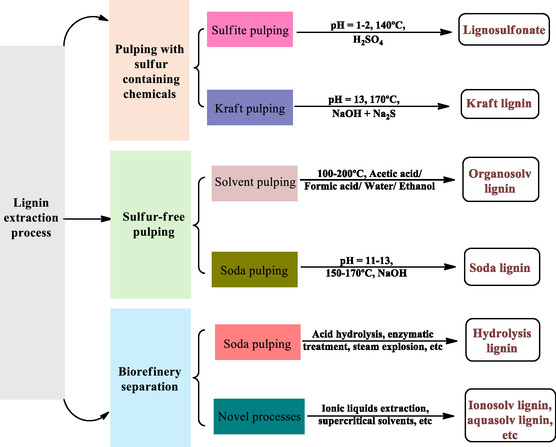
Lignin extraction processes and their products (drawn by authors after adaptation with permission.^[^ ^49^ ^]^ Copyright 2023, Royal Society of Chemistry).

Lignin content and structure in plants depend highly on the botanical origin and the extraction process used to recover it. Initially, lignin was recovered as a by‐product in the paper industry or through hydrolysis, by removing carbohydrates with strong acids such as sulfuric acid and hydrochloric acid.^[^ ^48^ ^]^ These methods led to considerable chemical modifications of the lignin structure. Subsequently, more advanced processes were developed that enabled lignin to be isolated and recovered separately from other by‐products. These newer, more precise extraction methods, such as organosolv, enzymatic hydrolysis, or ionic liquid extraction, help better preserve the lignin structure, paving the way for its use in various applications. Industrial processes yielding so‐called technical lignins can be divided into two categories: sulfur‐based and sulfur‐free pulping processes from the paper and pulp industry, which originally produced lignin as a by‐product (Kraft, sulfite, and soda processes), and biorefinery processes designed to convert biomass into multiple materials and chemicals. (**Figure** **3**). They allow for the isolation of lignins with high purity and reactivity (organosolv, ionic liquid, and hydrolysis).^[^ ^49^ ^]^

Among industrial pulping processes, the Kraft process is primarily used in the pulp and paper industry; it accounts for about two‐thirds of the world's virgin pulp production and over 90% of chemical pulp.^[^ ^50^ ^]^ The Kraft process produces a large amount of lignin as a by‐product; most of this lignin is not isolated for commercial use. Instead, it is typically burned for energy recovery within the pulping process itself.^[^ ^50^ ^]^ This process involves soaking wood with a solution called “white liquor,” composed of sodium hydroxide (NaOH) and sodium sulfide (Na_2_S).^[^ ^49^, ^51^ ^]^ The wood chips are then heated to a temperature that can reach 180 °C. During this heating step, bonds between the wood polymers are broken by alkaline hydrolysis, allowing the wood to be transformed into pulp that can be recovered by filtration, while the lignin and hemicelluloses are dissolved into a “black liquor.” Finally, lignin is extracted from the black liquor using additional processes, such as acid precipitation, which effectively precipitates it by lowering the pH to about 2.5. In Kraft lignin, the lignin is fragmented, contains low amounts of *β*‐*O*‐4 linkages, has a sulfur content of 1% to 3%, and is highly condensed. It is regularly used as a fuel in factories for energy and chemical recovery, and only a small portion of it is recovered for other applications.^[^ ^49^, ^51^ ^]^

Lignosulfonates, derived from the sulfite pulping processes, are the most commercially produced and widely available type of lignin.^[^ ^52^ ^]^ The global lignosulfonates market is projected to grow from USD 12 billion in 2022 to USD 1.4 billion by 2027, with a compound annual growth rate (CAGR) of 3.6% during that period.^[^ ^52^ ^]^ This growth is driven by increasing demand for lignosulfonates in various industries, including concrete additives, animal feed binders, and oil well additives. Borregaard, a major producer based in Norway, plays a significant role in the lignosulfonate market. Sulfite pulping involves producing pulp using a cooking liquor containing sulfite (SO_3_^2^^−^) or bisulfite (HSO_3_^−^) ions, along with different cations, which react with lignin through sulfonation at a temperature between 120 and 180 °C under high pressure to produce water‐soluble lignosulfonates (salts of lignosulfonic acids).^[^ ^53^ ^]^ The process generally occurs in an acidic environment, with a pH ranging from 2 to 5, but it can also take place in an alkaline pH. Lignosulfonates can be recovered by filtration and have a sulfur content of 2.1% to 9.4%.^[^ ^49^ ^]^ In contrast, soda pulping is a sulfur‐free process that yields lignin with low hemicellulose content. It uses sodium hydroxide (NaOH) at temperatures between 140 and 170 °C under high pressure (6 to 10 bar)>, producing lignin that is distinct from Kraft and sulfite lignins.^[^ ^49^ ^]^

Organosolv processes were also designed initially for paper manufacturing but later shifted towards lignin extraction. This method uses different combinations of organic solvents such as ethanol/water (Alcell process).^[^ ^54^ ^]^ This process allows for the separation of lignin and hemicelluloses from cellulose, which remains solid, while the lignins are precipitated from the spent liquor. The structure of organosolv lignin is closer to that of native lignin, and contains no sulfur.^[^ ^49^ ^]^ Some organosolv lignins have less than 1% carbohydrate content, while others contain high amounts of carbohydrates, depending on the efficacy of the extraction process.^[^ ^49^ ^]^

Several other biomass processing methods have also been developed specifically to extract lignin. Among them, ionic liquids (ILs) are increasingly being explored as efficient solvents for lignin extraction, offering a sustainable and effective alternative to traditional pulping methods.^[^ ^55^ ^]^ ILs can selectively dissolve lignin from the lignocellulosic matrix while preserving its structure, making it possible to recover high‐purity lignin.^[^ ^56^ ^]^ Their tunable chemical properties allow ILs to be customized for specific biomass components, enhancing their versatility in various applications. In recent years, several start‐ups and industrial companies have begun adopting ionic liquid‐based technologies in biorefineries to produce lignin for high‐value applications such as biofuels, polymers, and specialty chemicals. The ability to recycle ILs and the use of mild process conditions further contribute to the environmental and economic benefits of this approach.

Finally, enzymatic hydrolysis has emerged as a viable method for lignin extraction, offering the advantage of selectively breaking down cellulose and hemicellulose while maintaining lignin's structural integrity.^[^ ^57^ ^]^ This method employs specific enzymes, such as cellulases or lignin‐degrading enzymes (e.g., laccases or peroxidases), to selectively degrade biomass polysaccharides and disrupt lignin‐carbohydrate complexes. By carefully optimizing reaction conditions, these enzymes facilitate the release of lignin with minimal structural alterations, ensuring its integrity for subsequent applications.^[^ ^58^ ^]^ In contrast to conventional chemical extraction methods that often involve harsh conditions, enzymatic hydrolysis utilizes milder temperatures and pH ranges, presenting a more ecofriendly alternative for biomass processing.^[^ ^57^ ^]^ The growing emphasis on sustainable biorefineries has led to increased adoption of enzymatic hydrolysis by emerging companies, particularly for lignin recovery in the production of bio‐based materials and advanced products.^[^ ^49^ ^]^ This trend underscores the method's potential as a viable solution for industrial‐scale applications in the biorefinery sector.

The characteristics of lignin strongly depend on the extraction method chosen, with each process conferring unique properties to the lignin in terms of chemical structure, purity, and reactivity.

### 2.3 Lignin Chemical Modification Procedures

**Figure 4:**
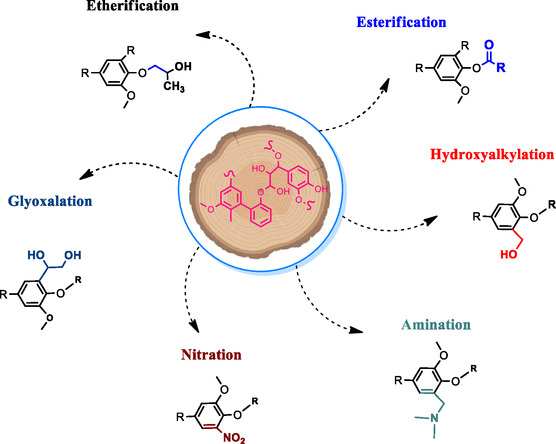
Overview of the most common lignin modifications in wood adhesives.

Lignins contain many functional groups, such as hydroxyl and methoxyl, making it a promising candidate for advanced applications. However, its complex structure presents challenges, such as chemical rigidity, brittleness, and poor solubility in solvents. Several chemical modification methods have been suggested to address these challenges, enhancing reactivity, reducing brittleness, and improving solubility.^[^ ^59^ ^]^ These include increasing the reactivity of hydroxyl groups and introducing new functional groups. This review highlights these modifications aimed at improving lignin applications in various products. **Figure** **4** presents common chemical modifications such as hydroxyalkylation, esterification, etherification, nitration, amination, urethanization, and glyoxalation, which are briefly outlined in the following subsections.

#### 2.3.1 Hydroxyalkylation

Hydroxyalkylation is a chemical modification that has been used to introduce new chemical sites in lignin structure (Figure 4). It involves adding hydroxymethyl (‐CH_2_OH) groups to lignin through a reaction with FA under alkaline conditions.^[^ ^60^ ^]^ This process boosts the number of reactive hydroxyl groups on lignin, which can then undergo further modifications. Typically conducted at temperatures ranging from 60–90 °C for 1–3 h, the reaction employs FA due to its high reactivity. Alternatively, paraformaldehyde can be used, albeit requiring a longer release time for the FA monomers.^[^ ^61^, ^62^ ^]^ Following the reaction, the hydroxymethylated lignin is separated through acidification and washing. This modification enhances lignin's reactivity and thermal stability, rendering it valuable in PF adhesive development.^[^ ^63^ ^]^

##### 2.3.1.1 Hydroxymethylation (or Methylolation)

Hydroxymethylation is a specific type of hydroxyalkylation that involves treating lignin with FA under alkaline conditions, thereby introducing hydroxymethyl groups into its structure. This modification enhances lignin's reactivity and thermal stability, expanding its applicability in polymer synthesis and composites.^[^ ^61^ ^]^ Researchers have observed that hydroxymethylation boosts lignin's reactivity by enabling its involvement in reactions previously inaccessible. For instance, hydroxymethyl groups can establish ether bonds with phenol, a key component in the production of lignin‐based PF resin adhesives. This enhancement in reactivity consequently improves mechanical strength, heat resistance, and overall performance of lignin‐based PF resin adhesives.^[^ ^63^ ^]^

##### 2.3.1.2 Phenolation

Another method to enhance lignin reactivity involves phenolysis or phenolation, where lignin is modified by reacting with phenol in organic solvents like methanol or ethanol under acidic conditions.^[^ ^64^ ^]^ This process is often used to modify lignosulfonates, aiming to increase phenolic group content and enhance the reactivity of the lignin structure.^[^ ^65^ ^]^ Primarily utilized in the synthesis of PF resins, this chemical modification entails pretreating lignin with phenol to enable its subsequent reaction with FA.^[^ ^66^ ^]^ This two‐step process is distinct from the methylolation process described earlier, as it introduces phenol groups to the lignin structure before FA reaction, whereas methylolation involves direct reaction of lignin with FA.

In summary, hydroxyalkylation effectively adds diverse functional groups (such as hydroxyalkyl or phenol groups) to the lignin structure. This process paves the way for subsequent chemical modifications and the creation of enhanced lignin‐based materials with added value.^[^ ^61^, ^66^ ^]^

#### 2.3.2 Esterification

Esterification stands out as a straightforward technique for modifying lignin's hydroxyl groups, achieved through their reaction with carboxylic acids, acyl chlorides, or acid anhydrides. This reaction, often involving condensation or ring‐opening polymerization, yields lignin‐based polyester networks, with water, hydrochloric acid, or carboxylic acid as by‐products.^[^ ^64^ ^]^ Enhancing lignin's compatibility and thermal stability, esterification renders it suitable for various applications like polymer blends, 3D printing inks, and packaging materials.^[^ ^62^, ^67^ ^]^ Conventional esterification protocols employing anhydrides or acyl chlorides are associated with certain sustainability limitations.^[^ ^62^ ^]^ However, emerging ecofriendly methodologies leveraging organic acids as dual solvents and reactants offer promising avenues to mitigate these concerns. Adjusting the extent of esterification and the nature of introduced ester functionalities is feasible through modulation of parameters such as reaction time, temperature, and the nature of the organic acid employed.^[^ ^62^ ^]^

While esterification has not been directly applied to UF resin systems, it represents a promising method for modifying lignin to enhance its compatibility with various polymer matrices. Similar lignin modification strategies, such as hydroxymethylation and reaction with maleic anhydride, have successfully improved lignin's utility in UF resin applications. The principles of lignin modification for improved polymer compatibility could potentially be extended to esterified lignin in UF resin systems, opening avenues for future research.

##### 2.3.2.1 Sulfation

On the other hand, direct **sulfation wish is an esterification process** allows the attachment of sulfate groups (‐SO_3_^−^) via ester bond (C‐O‐S) to lignin by treating it with sulfuric acid.^[^ ^68^ ^]^ This can be accomplished by reacting lignin with sulfuric acid or other sulfur‐containing reagents.^[^ ^69^ ^]^

##### 2.3.2.2 Phosphorylation

Another method of esterification, known as **phosphorylation**, involves the incorporation of phosphate groups (‐*O*‐PO_3_H_2_) into the lignin structure.^[^ ^70^, ^71^ ^]^ Since the 1980s, hydroxyl groups within lignin have been extensively modified using various chloro‐phosphorus‐containing compounds via the Williamson reaction for diverse applications.^[^ ^72^ ^]^ Additionally, phosphoric acid, hypophosphoric acid, phosphate salts, and phosphoric anhydride are also utilized as phosphorus agents to achieve this modification.^[^ ^72^, ^73^ ^]^

##### 2.3.2.3 Sulfomethylation

This process is not an esterification but involves the addition of both sulfonic acid (‐SO_3_H) and methyl (‐CH_3_) groups to the lignin structure.^[^ ^69^ ^]^ Typically, this process entails reacting lignin with FA and sodium sulfite or bisulfite under alkaline conditions in water. During this reaction, FA adds a hydroxymethyl group to the softwood Kraft lignin, which is then substituted by sulfite to form methylene sulfonate groups.^[^ ^68^ ^]^

#### 2.3.3 Etherification

Among the variety of processes employing lignin's hydroxyl groups, etherification emerges as notably facile due to its favorable reaction conditions and choice of reactants. This process typically involves the use of alkyl halides, epoxides, or similar reagents under basic conditions, facilitating the formation of ether linkages. Alkyl halides, particularly chloroalkanes, are commonly used for their heightened reactivity.^[^ ^66^ ^]^ In general, etherification is a useful chemical modification technique that transforms the hydroxyl groups (phenolic and aliphatic) in lignin into ether linkages by reacting with chemicals like epoxides or alkyl halides.^[^ ^65^ ^]^ This modification changes the reactivity and characteristics of the lignin matrix by introducing an additional ether (‐*O*‐) connection.

##### 2.3.3.1 Oxypropylation

Oxypropylation is one of the most popular etherification methods for lignin modification.^[^ ^74^ ^]^ This process primarily aims to convert the phenolic hydroxyl (OH) groups in lignin to aliphatic OH groups, making lignin more suitable for polyurethane (PU) applications.^[^ ^75^ ^]^ Oxypropylation facilitates the transformation of insoluble, solid lignin into highly soluble polyols that are compatible with a wide range of organic solvents. The reaction involves the interaction of propylene oxide (PO) with oxianions, which are produced when lignin's OH groups react with an alkaline catalyst, typically KOH.^[^ ^66^ ^]^ This modification not only enhances lignin's solubility but also improves its reactivity and compatibility with PU systems, opening up new possibilities for lignin utilization in polymer applications.

##### 2.3.3.2 Silylation

Silylation, an alternative form of etherification, is relatively underrepresented in current literature. It entails the introduction of silyl ether groups (‐*O*‐Si‐) onto the lignin molecular structure.^[^ ^76^, ^77^ ^]^ Typically, silylation reactions involve the treatment of lignin with silylating agents such as trimethylsilyl chloride (TMSCl), divinyldisilazane (DVDZ), or hexamethyldisilazane (HMDS). This approach is proposed as an effective means to modify Kraft lignin chemically, enhancing its thermal stability and solubility characteristics, thereby broadening its potential applications as a renewable aromatic polymer.^[^ ^78^, ^79^ ^]^

#### 2.3.4 Amination

Amination is a different way to modify lignin. It includes adding amine groups (‐NH_2_, ‐NHR, or ‐NR_2_) to the lignin structure. The predominant method for aminating lignin is via the Mannich reaction, where lignin reacts with amines and FA.^[^ ^80^ ^]^ Recent investigations frequently employ diethylamine and FA under varying reaction conditions for lignin amination.^[^ ^66^ ^]^ The Mannich reaction proceeds through the interaction between a carbon possessing high electron density and an iminium ion generated from FA and an amine. Consequently, an aminoethyl group can be introduced at the ortho position of a phenolic hydroxyl group.^[^ ^65^, ^66^ ^]^ Amination improves lignin's compatibility, dispersibility, and solubility, making it better suited for blending into coatings, polymer blends, and other products.^[^ ^81^, ^82^ ^]^

#### 2.3.5 Nitration

Nitration presents an alternative method to introduce novel functional groups into lignin, enabling its modification in nonaqueous solvents using nitrating agents like nitric acid with acetic anhydride, nitric acid in concentrated acetic acid, and fuming sulfuric acid to incorporate nitro (‐NO_2_) groups into its framework.^[^ ^80^, ^83^ ^]^ The resultant product typically appears as a yellow to brown amorphous powder, with a molecular weight ranging from 600 to 2000 Da and a nitrogen content varying between 6% and 7%.^[^ ^65^ ^]^ Furthermore, nitration can induce demethylation of Kraft lignin, leading to increased methanol and nitro group formation.^[^ ^84^ ^]^ This process serves as a valuable means to depolymerize and chemically alter the lignin structure, thus opening new avenues for its utilization.^[^ ^83^ ^]^ In essence, the nitration of lignin utilizing nitric acid and related reagents represents a significant chemical transformation, imparting alterations to the structure and properties of this renewable aromatic polymer, thereby holding promise for diverse applications in materials and chemicals.

#### 2.3.6 Glyoxalation

Most recent literature, including reviews on lignin, and book chapters, overlooks glyoxalation as a method for chemically modifying lignin.^[^ ^63^, ^64^, ^65^, ^66^, ^80^ ^]^ Glyoxalation entails the reaction of lignin with the dialdehyde compound glyoxal (HCHO‐CHO). Typically conducted in an alkaline environment, the reaction is facilitated by adjusting the pH to ≈12.5 using a base such as sodium hydroxide (NaOH).^[^ ^85^ ^]^ The process involves mixing lignin with water, adding glyoxal solution, and heating the reaction mixture to around 75 °C. Through this reaction, glyoxal reacts with the hydroxyl groups present in lignin, introducing aldehyde and acetal functionalities.^[^ ^85^, ^86^ ^]^ Glyoxalation, involving the addition of glyoxal as a dialdehyde, has been highlighted as a particularly promising method for modifying lignin (bagasse soda black liquor with pH = 13% and 40% solid content as a source of lignin) for use in wood adhesives.^[^ ^87^ ^]^ The heightened reactivity and improved compatibility of glyoxalated lignin underscore its potential as a renewable resource for developing sustainable, lignin‐based products.

To summarize, lignins can undergo significant chemical changes known as glyoxalation, which add acetal and aldehyde functionalities to the lignin structure. This modification changes the lignin's characteristics and increases its potential applications in creating lignin‐based materials with added value.

## 3 Advancements in Lignin‐Based UF Formulations

As known, urea‐formaldehyde (UF) resin, a synthetic polymer, is synthesized through a two‐step process: first, the addition of urea and FA to form methylolurea, followed by the condensation of methylolurea molecules.^[^ ^88^ ^]^ UF resin holds a significant position in adhesive production, constituting approximately half of the annual output, with global production estimated at 5 million tons.^[^ ^19^, ^59^ ^]^ This resin is pivotal in the fabrication of wood‐based panels like particleboard and MDF, and over 90% of these panels utilize UF adhesives.^[^ ^19^, ^24^ ^]^ Consequently, a substantial portion of UF adhesives manufactured globally is consumed by this sector.^[^ ^89^ ^]^ UF resin boasts numerous advantageous properties, such as high reactivity, rapid curing, postcure transparency, favorable processability, cost‐effectiveness, and superior bonding performance, cementing its status as a primary adhesive in the wood industry for interior applications.^[^ ^59^ ^]^ However, challenges persist, notably in terms of formaldehyde emissions (FE) and water resistance^[^ ^24^, ^59^, ^87^ ^]^ constraining its widespread application. Over the past decade, extensive research has explored various strategies to address these limitations, including incorporating or modifying UF resin through lignin utilization.^[^ ^59^, ^90^ ^]^

At present, the pulp and paper industry yields over 50–70 million tons of lignin each year, and it is estimated to increase by 225 million tons by 2030^[^ ^91^ ^]^ with the major part of that quantity primarily utilized as fuel, leaving only a mere 4.5% for the development of high‐value products.^[^ ^92^ ^]^ In this context, the subsequent sections will spotlight recent endeavors concerning the utilization of lignin from various origins, both with and without chemical modification, in the advancement of UF adhesives. **Table** **1** succinctly encapsulates the impact of lignin application, whether as a replacement or introduced during synthesis, on the characteristics of wood adhesives. The findings reveal significant enhancements in all optimal formulations incorporating modified or unmodified lignin materials, underscoring their affirmative role in the evolution of UF adhesives for wood bonding applications.

**Table 1:** Characteristics, optimal amount, chemical modification, and main results of lignin‐based UF adhesives.

Lignin types	Chemical modification	Optimal amounts	Type of wood product	Role played	Main results	Formaldehyde emission	Ref
Commercial sulfonated Kraft lignin	None	30%	No application	Partially replace UF resin	Improved thermal stability of the UF resin after lignin addition	No experimental data	[94]
Amphiphilic sodium lignosulfonate	None	20%	Plywood	Addition during UF synthesis, partially replacing urea	‐Significant influence in the colloidal morphology of UF resins ‐The shear strength was similar to reference panel	Slight increase in FA release, lower than the requirements for FA emission of E0 grade plywood	[96]
Ammonium lignosulfonate (ALS)	None	6% ALS + 4% UF	HDF	Replacement of UF resin	‐MOE, MOR, and IB increased by 33.47%, 45.90%, and 38.46% ‐TS and WA decreased by 28.55% and 22.33%	FE decreases from 6.4 to 1.7 mg/100 × g with a reduction rate of 73.43%	[97]
Ammonium lignosulfonate (ALS)	None	10% ALS + 3% UF	HDF	Replacement of UF resin	‐MOE, MOR, and IB increased by 29%, 31%, and 17% ‐TS and WA decreased by 29.5% and 21%	FE decreases from 4.3 to 1.0 mg/100 × g with a reduction rate of 76.74%	[98]
Alkali lignin from *Solanum elaeagnifolium* Cavanilles Weeds	None	15%	Particleboards	Replacement of UF resin	MOE, MOR, and IB increased by 36.78%, 26.66%, and 19.56%	FE decreases from 0.85 to 0.61 mg/100 × g with a reduction rate of 28.23**%**	[100]
Alkali lignin from bagasse	None	10%	Particleboard	Replacement of UF resin	Remarkable increase in MOE, MOR, IB by 26.65%, 31.25%, and 23.61%, respectively, exceeding the EN 312 (P4 and P2)	FE decreases from 3.95 to 3.1 mg/100 × g with a reduction rate of 21.51%	[99]
Soda and Kraft lignins from bagasse	None	20%	Particleboard	Addition during UF and PF synthesis, partially replacing urea/phenol	‐Soda lignin exhibited higher phenolic hydroxyl groups and lower glass transition temperature. ‐Panels bonded with soda‐based resin yielded lower WA as well as higher strength	Panels bonded with soda‐based resin yielded lower WA and FE	[101]
Kraft lignin and lignosulfonate	None	‐5% for Kraft and 10% for lignosulfonate	Particleboard	Partially replace UF resin	‐The lowest TS and WA of 71.16% and 129.17% were determined for the boards with lignosulfonate. ‐MOR and MOE increased by 43% and 2.5%	None	[102]
Magnesium and sodium lignosulfonates (LS)	None + PMDI as a crosslinker	30%	Particleboard	Replacement of UF resin	‐The addition of both lignin deteriorated the water absorption and thickness swelling of boards. ‐ Boards with neat UF showed the best mechanical properties	Na‐LS: UF bonded boards had a lower formaldehyde content (FC) than Mg‐LS: UF and UF‐bonded boards as control	[103]
Soda bagasse lignin	Ionic liquid modification	20%	Plywood	Addition during UF synthesis, partially replacing urea	‐Faster gel time compared to glyoxalated lignin ‐Remarkable decrease in WA ‐Slight decrease in SS from 1.93 MPa to 1.32 MPa	FE decreases from 5.4 to 3 mg/100 × g with a reduction rate of 80%	[106]
Soda bagasse lignin + pMDI	Ionic liquid modification	6% of pMDI	Plywood	Addition of Na‐ pMDI as an additive to the previously prepared GLUF resin	‐The addition of pMDI increases the reactivity of the resin resulting in a shorter gelation time and a reduction in curing temperature ‐The SS highly improved in dry and wet states by 41.02% and 41.42%	FE decreases from 3.5 to 2.9 mg/100 × g with a reduction rate of 17.14%	[107]
Soda bagasse lignin	Glyoxalation	15%	Plywood	Addition during UF synthesis, partially replacing urea (GLUF resin)	‐Curing process shifted to lower temperature ‐Water absorption decreases from 48% to 30% ‐Shear strength decreased from 1.93 MPa to 1.22 MPa	FE decreases from 4.3 to 3.2 mg/100 × g with a rate of 34.37%	[87]
Lignin	Hydroxymethylation + the reaction with maleic anhydride	‐5% ‐7.5%	‐Plywood ‐MDF		‐Improvement in the SS of plywood (increase from 1.04 to 1.72 MPa), ‐Remarkable enhancement in the MOE, MOR, IB, WA and TS of MDF.	‐Plywood FE decreases from 1.08 to 0.19 mg/100 × g with a reduction rate of 82.4% ‐ MDF FE decreases from 3.25 to 2.32 mg/100 × g with a reduction rate of 28.6%	[108]
Lignosulfonate (LS‐OB)	Oxidation by introducing oxygen at 150 °C for	40%	Particleboard	Partially replace UF resin	‐LS‐OB, positive effect on the curing of resin. ‐Improved IB from 256 PSI to 280 PSI ‐Reduction in TS from 30 to 21%	No experimental data	[93]
Kraft lignin	Phenolation	20%	Particleboard	Addition during UF synthesis, partially replacing urea	‐Decrease in the crystallinity of UF ‐No significant differences in IB compared to neat UF resin	FE decreases from 4.3 to 2.8 mg/100 × g with a reduction rate of **34.37%**	[105]
Soda bagasse lignin + Na‐MMT	Glyoxalation	15% of GL + 1.5% of Na‐MMT	Particleboard	Addition of Na‐MMT as additive to the previously prepared GLUF resin	‐Nanoclay addition improved the physicochemical of the adhesive ‐MOE, MOR, and IB increased by 13%, 15%, 15.7% and 40% ‐TS and WA decreased by 28% and 11%	FE decreases from 3.5 to 3.1 mg/100 × g with a reduction rate of 11.42%	[104]
Thick spent sulfite liquor (contains 58% of lignosulfonated lignin)	Hydroxymethylation	10%	Particleboard	Addition during UF synthesis, partially replacing urea	No positive effect observed in the SS, IB, MOE and MOR	FE decreases from 5.8 to 4.3 mg/100 × g with a reduction rate of 25.86%	[109]

### 3.1 Unmodified Lignin‐Based UF Adhesives

Historically, lignin has been chiefly employed to substitute phenol in PF resins.^[^ ^59^ ^]^ However, in recent years, there has been growing interest and research into incorporating lignin in UF resins, either as a partial replacement for the entire resin or as a substitute for a fraction of urea during resin synthesis. Chen et al.^[^ ^93^ ^]^ first introduced lignin as a partial replacement for UF in 1994. Later, lignin incorporation during UF synthesis was introduced by Kordkheili et al.^[^ ^87^ ^]^ in 2015.

**Figure 5:**
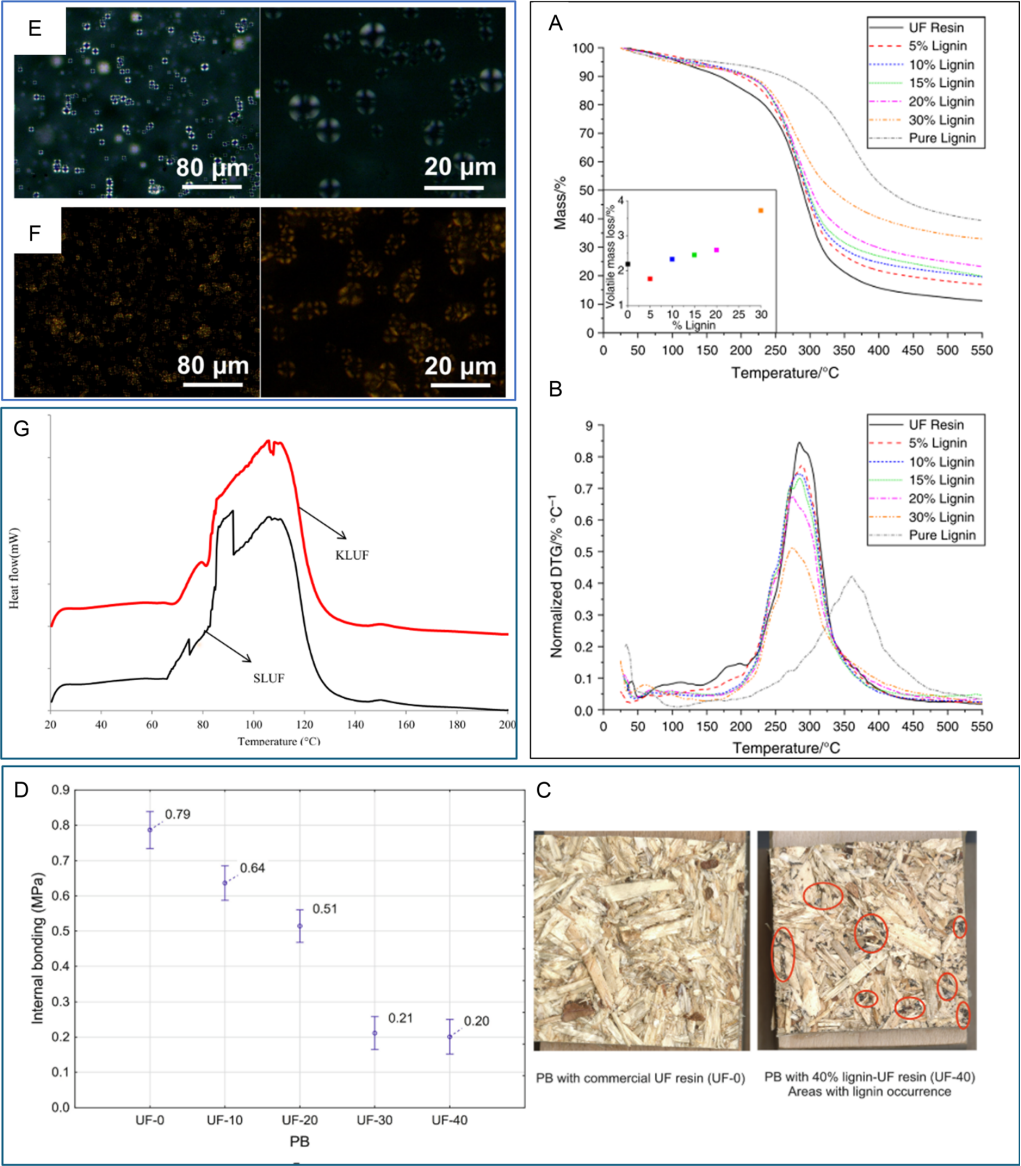
A) TGA and DTG. B) Curves of neat UF resin and formulations incorporating 5%, 10%, 15%, 20%, and 30% sulfonated Kraft lignin. C) Sample deformation images of particleboards. D) Internal bond strength of panels fabricated using neat UF and UF formulations modified with various lignin contents. Polarized microscopy images of aged resin samples stored at room temperature: E) UF and F) UF with lignosulfonate. G) DSC curves showing curing behavior of UF resins synthesized using soda lignin and Kraft lignin. All figures were reproduced with permission.^[^ ^94^, ^95^, ^96^, ^101^ ^]^ Copyright 2019, EPA; 2024, Elsevier; 2020, Elsevier; 2023, Elsevier.

In 2019, a study conducted by Natarelli et al.^[^ ^94^ ^]^ investigated the utilization of 30% of sulfonated Kraft lignin, obtained from industrial hardwood (Eucalyptus spp), as a partial replacement for UF resin (70% of solid content) and its consequent effects on thermal degradation and stability. Through a thermogravimetric analysis (See **Figure** **5**A and B), the researchers examined blends of UF resin with a commercial sulfonated Kraft lignin to assess the impact of lignin addition on the thermal behavior of the adhesive system. Their findings shed light on the enhanced thermal stability achieved with lignin incorporation, despite observed changes in activation energy. Moreover, the study provided insights into reducing FA emissions possibly due to lignin's FA scavenging properties, its role as a physical barrier in the resin matrix, the overall reduction in UF content, or alterations in the curing and degradation processes of the adhesive system, suggesting a promising avenue for improving the environmental performance of UF adhesives through lignin utilization. In a different work, Nemec and his colleagues^[^ ^95^ ^]^ precipitated Kraft lignin from commercial hardwood black liquor and incorporated it into UF resin for particleboard production, using up to 40% lignin content. The panels were pressed at a temperature of 140 °C for 5 min with a pressure of 3.5 MPa. The results showed that incorporating 40% Kraft lignin significantly decreased the mechanical properties, with bending strength dropping from 13.15 MPa to 4.62 MPa, modulus of elasticity (MOE) decreasing from 1858 MPa to 995 MPa, and internal bond strength (IB) reducing from 0.79 MPa to 0.20 MPa (See Figure 5C and D). Additionally, the physical properties exhibited substantial increases, with thickness swelling (TS) rising from 36.63% to 77.53%, indicating a negative effect. Most of the produced variants did not meet the standardized criteria for particleboards defined by EN 312. However, the particleboard variants with 10% and 20% lignin in UF resin meet the requirements for P2 boards (suitable for interior use) in terms of internal bonding, and the variant with 10% lignin in UF resin also meets the bending strength requirements for P2 boards.

Another approach by Gao et al.^[^ ^96^ ^]^ investigated the impact of lignosulfonate addition to the UF resin formulation for plywood application, where the panels were hot‐pressed at 12 MPa between 120–125 °C for 60 s per millimeter of thickness. They found that lignosulfonate greatly influenced UF resin colloidal morphology, leading to more uniform and smaller microspheres when added during initial resin formation under basic conditions (see Figure 5E and F). Structural analysis revealed lignosulfonate's role as a surfactant, enhancing electrostatic repulsion among resin particles to prevent aggregation. They also found that lignosulfonate greatly improved UF resin storage stability, extending it from 30 to 200 days. Despite a slight reduction in curing rate, lignosulfonate enhanced thermal stability without compromising adhesive performance, showing comparable shear strength and free FA emission to resins without lignosulfonate (see Table 1).

In a separate work, Antov et al.^[^ ^97^ ^]^ investigated the use of ammonium lignosulfonate (ALS) in low amount as an environmentally friendly additive of UF resin for producing high‐density fiberboard (HDF) panels. The panels were hot‐pressed at 220 °C using a four‐stage schedule: i) 45 MPa for 15 s, ii) 1.2 MPa for 15 s, iii) 0.6 MPa for 30 s, and iv) 1.8 MPa for 30 s. They manufactured HDF panels with 4% of UF resin content and varying ALS addition levels from 4% to 8% based on dry wood mass. The resulting panels demonstrated satisfactory physical and mechanical properties suitable for load‐bearing applications, with notably low free FA emission levels (ranging from 2.0 to 1.4 mg/100 g), meeting “E0" and super “E0" emission grade requirements. Overall, the study concluded that addition of ALS, as a bio‐based additive, can effectively enhance UF adhesive formulations for wood‐based panels, providing environmental benefits in industrial applications. The incorporation of lignin positively impacted the mechanical properties, water resistance, and FA emissions of elaborated wood panels.

The same research group conducted a different study focused on investigating the potential of producing ecofriendly HDF panels using hardwood fibers bonded with UF resin and ALS additive.^[^ ^98^ ^]^ The HDF panels were prepared in the laboratory with a very low UF gluing factor (3%) and varying ALS content (6% to 10% based on dry fibers), the hot‐pressing process was at 200 °C, and a press factor of 30 s.mm^−1^ using a four‐stage pressing regime: 4.5 MPa for 20 s, then gradually reduced to 1.2 MPa, followed by 0.6 MPa, and finally 1.8 MPa. The HDF panels demonstrated satisfactory physical and mechanical properties suitable for applications in humid conditions (see Table 1). Notably, the FA content of the panels was exceptionally low, ranging between 0.7–1.0 mg/100 g, equivalent to the FA release of natural wood. This study highlights the effectiveness of the ALS additive in producing FA‐free HDF panels with desirable properties, presenting a significant advancement over previous research.

In a complementary study, Moubarik's group^[^ ^99^ ^]^ investigated the potential of utilizing alkali lignin as a sustainable alternative in UF adhesive formulations for particleboard applications, using a total pressing time of 7 min at 190 °C with maximum pressure of 23 kg cm^−2^. The first study focused on lignin isolation from sugar industry by‐products, particularly bagasse and molasses beet, through alkali treatment. By partially substituting up to 20% of the solid content of UF resin with lignin, the researchers observed remarkable improvements in mechanical properties. Specifically, adding 15% of bagasse lignin resulted in increases in bending MOR, MOE, and IB of 26.65%, 31.25%, and 23.61%, respectively, compared to reference panels (MOR = 16 MPa, MOE = 2544 MPa, and IB = 0.40 MPa). These enhancements exceeded the EN 312 (P4 and P2) standards, indicating the potential of lignin as an effective additive. Furthermore, FE decreased from 3.95 to 3.1 mg/100 g, with a reduction rate of 21.51%, emphasizing the ecofriendly advantages of the lignin‐UF adhesive formulation. In contrast, the second study explored lignin isolation from *Solanum elaeagnifolium* Cavanilles (SE‐Cav).^[^ ^100^ ^]^ Incorporating 15% of this lignin into UF resin formulations for particleboard production also resulted in notable improvements, with bending MOE, MOR, and IB increasing by 36.78%, 26.66%, and 19.56%, respectively. Additionally, FE decreased from 0.85 to 0.61 mg/100 g, with a reduction rate of 28.23%. When comparing the two studies, the mechanical property enhancements from SE‐Cav lignin (MOE increase of 36.78% and IB increase of 19.56%) were generally higher than those observed with bagasse lignin. Furthermore, the reduction in FE was more significant with SE‐Cav lignin, suggesting it may be a more effective additive in UF adhesive formulations. However, critical considerations such as scalability, sustainability of lignin sourcing, and practical applicability warrant further investigation in future research endeavors to ensure the viability of these promising alternatives in industrial applications.

In a related work, led by Pizzi,^[^ ^101^ ^]^ up to 40% of lignin derived from the treatment of sugarcane bagasse through a soda and Kraft pulping process was incorporated into FA‐based resins (PF and UF (solid content fixed at 52%)) for the improvement of particleboard properties. The boards were hot‐pressed at a pressure of 35 MPa and temperature set at 160 °C for lignin‐urea‐formaldehyde (LUF) and 180 °C for lignin‐phenol‐formaldehyde (LPF). In general, the UF resins with 20% soda lignin showed a shorter gel time, indicating potential advantages in processing. Moreover, the curing temperatures of soda lignin resins (20%) were lower than those made from 20% Kraft lignin (see Figure 5G). More importantly, in terms of water absorption, FE, flexural strength, and internal bonding strength, the properties of particleboards bonded by Soda lignin‐based resins were better than those bonded by Kraft lignin‐based resins. Additionally, the study revealed that the mechanical properties of particleboards bonded with 20% LPF resins could be significantly enhanced by increasing lignin content, while the opposite trend was observed for LUF resins.

Recently, Madyaratri et al.^[^ ^102^ ^]^ investigated the effect of two lignin additions on the properties of particleboard made from Areca leaf sheaths, a type of agricultural waste, using UF resin. The mixtures were heated under pressure for 10 min at 150 °C. In their study, two types of lignins were employed, Eucalyptus Kraft lignin and commercial sodium lignosulfonate from Sigma‐Aldrich, and both were incorporated into the UF resin to examine their impact on the physical, mechanical, and fire‐resistant properties of the boards. The researchers applied multiple washing treatments to these lignins to enhance their performance. Kraft lignin was washed three times, while lignosulfonate underwent three and five washing treatments. Boards bonded with 5% of these modified lignins were then evaluated for key performance parameters. Their findings revealed that the highest board density was achieved with UF resin mixed with Kraft lignin, which had been washed three times. For moisture‐related properties, the lowest moisture content was observed in boards bonded with three‐times‐washed lignosulfonate. In terms of dimensional stability, boards containing commercial lignin exhibited the lowest thickness swelling, while those bonded with five‐times‐washed lignosulfonate showed the lowest water absorption. The mechanical performance of the boards improved significantly with the washing treatments. Particleboards bonded with lignosulfonate that had undergone five washing treatments exhibited the highest bending MOR and MOE, reaching values of 113 MPa and 10 663 MPa, respectively. More importantly, all the lab‐made boards showed good fire resistance according to the UL‐94 standard; the lowest weight loss was recorded for the boards fabricated with LS five washing treatments. This work underlines the feasibility of improving the fire resistance of the board with the addition of lignin‐based fire retardants to develop a lignocellulosic composite material while reducing its carbon footprint.

The utilization of other materials as crosslinkers in combination with untreated lignin was introduced by the team of Savov in 2021.^[^ ^103^ ^]^ In their work, authors reported the possibility of using magnesium and sodium lignosulfonates (LS) in particleboard production. The mattress was hot‐pressed at 25 MPa and 200 °C for 600 s. They used LS both as a sole binder and in combination with UF resin, having polymeric 4,4'‐diphenylmethane diisocyanate (pMDI) as a crosslinker. The study assessed the effects of gradually replacing UF with magnesium lignosulfonate (MgLS) or sodium lignosulfonate (NaLS) on water absorption and tensile properties. Particleboards bonded with LS‐UF formulations, containing up to 30% LS, exhibited comparable internal bonding strength and modulus of rupture to the neat UF‐bonded particleboards. However, the LS‐UF formulations resulted in higher water absorption and thickness swelling, making them more susceptible to moisture compared to the UF‐bonded panels. Particularly, boards bonded with NaLS‐UF showed a lower formaldehyde content (FC) than those bonded with MgLS‐UF and UF boards. Although the addition of pMDI improved the mechanical properties of LS‐UF formulations, pMDI's high reactivity with water raised challenges in formulating stable water‐based adhesives. Despite these enhancements, pure UF‐bonded boards exhibited superior mechanical strength, confirming UF's dominant performance as a binder. However, LS‐bonded boards achieved substantially lower FC levels, meeting the stringent Super E0 emission standards.

When comparing the results across these studies, it becomes evident that the source and extraction method of lignin significantly affect the performance of UF adhesives. Several factors, including molecular weight, functional group content, and solubility, play critical roles in determining its reactivity and interaction with the UF matrix, which in turn impacts the adhesive's overall performance.

For instance, soda lignin, which contains more phenolic and methoxy groups, demonstrates better water resistance and curing behavior than Kraft lignin due to its enhanced reactivity and lower molecular weight, which improves dispersion in the resin. Similarly, lignosulfonates, with their sulfonate groups, improve resin dispersion, stability, and FE reduction. Alkali lignins derived from agricultural by‐products also show promising mechanical performance and emission reduction, thanks to their lower molecular weight and reactive hydroxyl groups, which promote better integration into the UF matrix. These variations in performance are mainly due to differences in lignin's molecular characteristics. High molecular weight lignins, such as Kraft lignin, form rigid structures that limit resin integration, while lower molecular weight lignins, like soda and alkali lignins, disperse more easily, improving adhesive performance. Functional groups such as phenolic and hydroxyl groups strengthen bonds with the UF matrix, enhancing mechanical properties and reducing FE. Lignin solubility also plays a crucial role in ensuring uniform dispersion, improving resin consistency, and curing behavior.

In conclusion, these studies highlight the importance of selecting the appropriate lignin source and extraction method to optimize the performance of UF adhesives. The molecular characteristics of lignin, such as molecular weight, functional group content, and solubility, must be carefully considered to achieve the desired balance between mechanical strength, water resistance, FE reduction, and overall adhesive performance. Future research should focus on understanding the specific interactions between different lignin types and the UF matrix to further optimize the use of lignin in adhesive formulations.

### 3.2 Chemically Modified Lignin‐Based UF Adhesives

Functionalizing lignin by introducing various chemical groups like phenols, glyoxal, and hydroxymethyl can significantly improve its physicochemical properties. These chemical modifications alter the molecular structure of lignin by increasing the number of reactive sites (e.g., hydroxyl or aldehyde groups), enhancing its ability to form covalent bonds with FA and urea through condensation reactions. These modifications improve the lignin's solubility, reactivity, and thermal stability, making it more suitable for specialized applications such as in adhesives, coatings, and composite materials.^[^ ^69^, ^80^ ^]^ For instance, hydroxymethylation introduces hydroxymethyl groups (‐CH_2_OH) that increase cross‐linking density in the UF resin matrix, while glyoxalation provides aldehyde functionalities that can cocondense with urea, forming methylene and methylene ether bridges similar to FA‐based reactions. By tailoring the functional groups, the performance characteristics of lignin can be optimized to meet specific industrial requirements.

In 1994, Chen and colleagues^[^ ^93^ ^]^ developed oxidized lignosulfonate by introducing oxygen at a partial pressure of 25 atm (101.3 kPa) into a 5% aqueous solution of lignosulfonate at 150 °C for a specific duration. This modified lignosulfonate from softwood was used as a partial substitute for UF adhesive to prepare particleboard panels, which were produced by hot‐pressing at 2.9 MPa and 180 °C for 3.5 min. The results showed that adding 40% lignosulfonate significantly improved the curing of the resin and the mechanical and physical properties of the particleboard. The internal bond strength increased from 256 PSI (1.7651 MPa) to 280 PSI (1.9305 MPa), and the thickness swelling was reduced from 30% to 21% compared to the reference panels. This improvement can be attributed to the higher content of carboxylic and phenolic groups formed during oxidation, which promotes a greater extent of cross‐linking with the UF matrix. However, using more than 40% lignosulfonate had detrimental effects, likely due to insufficient active groups in the lignosulfonate to fully react with the UF resin and form a complete 3D matrix.

In a series of studies, Younesi‐Kordkheili et al.^[^ ^87^, ^104^ ^]^ investigated the enhancement of UF resins using modified lignin and some additives for improved performance and reduced environmental impact. In one study, glyoxalated soda bagasse lignin was incorporated at 10%, 15%, and 20% at pH 7 during UF resin synthesis, replacing the second urea amount. The resulting resin was used for plywood production, where three layers were assembled and hot‐pressed at 120 °C under a maximum pressure of 1 MPa for 5 min.^[^ ^87^ ^]^ Generally, glyoxalation introduces dialdehyde groups that can effectively cross‐link with urea, enhancing the structural integrity of the resin. The obtained results demonstrated that the modified lignin‐based resins provided excellent shear strength for plywood panels, meeting international standards. Additionally, these panels exhibited reduced FE and water absorption compared to commercial UF adhesives (Table 1). Specifically, the UF resin with 15% glyoxalated lignin (GLUF) maintained lower water absorption and FE, with no significant differences in shear strength and physicochemical properties compared to the control UF resin. DSC analysis showed that the curing process of the GLUF resin occurred at lower temperatures than the UF resin. FTIR spectra revealed that the introduction of lignin reduced the proportion of C–N bonds in methylene linkages when urea was partially replaced by lignin or glyoxalated lignin. This suggests a substitution of traditional methylene bridges with alternative linkages involving lignin's functional groups. Furthermore, XRD analysis indicated a decrease in the crystallinity of the UF resins with the addition of glyoxalated lignin.

In a follow‐up study, the group optimized the GLUF resin by adding sodium‐montmorillonite (NaMMT) nanoclay at concentrations of 0.5%, 1%, and 1.5% for particleboard production. The boards were hot‐pressed at a pressure of 3.5 MPa and a temperature of 150 °C.^[^ ^104^ ^]^ The inclusion of 1.5% NaMMT improved mechanical strength, reduced water absorption, and further lowered FE. XRD results confirmed NaMMT intercalation within the resin, and DSC analysis showed accelerated curing with higher reaction enthalpy. FTIR analysis also indicated structural modifications in the GLUF resin due to nanoclay incorporation. NaMMT likely promotes better dispersion of reactive groups and acts as a nanoreinforcement, facilitating enhanced cross‐linking and mechanical performance.

In a subsequent investigation, they prepared **phenolated lignin** from Kraft black liquor to mitigate FE in particleboard production. The press plates were maintained at a temperature of 180 °C and a pressure of 25 bar.^[^ ^105^ ^]^ The lignin was modified through phenolation under acidic conditions, aligning with the synthesis environment of UF resins. Phenolation increases the number of phenolic hydroxyl groups on the lignin backbone, boosting its reactivity with FA via electrophilic aromatic substitution, similar to the PF reaction mechanism. Various concentrations of both unmodified and phenolated lignin (up to 20%) were incorporated. The optimized UF resin with 20% phenolated lignin reduced FE by 34.37%, from 43 mg/100 × g to 2.8 mg/100 g, without compromising internal bond strength or other physicochemical properties of the panels. This study identified phenolated lignin as an efficient bio‐based alternative for formaldehyde reduction in wood adhesives.

In a subsequent investigation, the group explored the use of 1‐ethyl‐3‐methylimidazolium acetate IL, a nontoxic organic reagent, to modify soda bagasse lignin for further improvement of UF resins in plywood production. Three layers were assembled and hot‐pressed at 120 °C under a maximum pressure of 1 MPa for 5 min.^[^ ^106^ ^]^ The modified and unmodified lignins (10%, 15%, and 20%) were introduced at pH 7 during UF resin synthesis to replace part of the urea content. The IL‐modified lignin resins demonstrated better performance than those with unmodified lignin, resulting in significantly lower water absorption with faster gel times. IL treatment likely disrupts lignin's recalcitrant structure, increasing solubility and reactivity by exposing or generating additional functional groups capable of participating in polycondensation reactions. Though the shear strength decreased slightly (from 1.93 MPa to 1.32 MPa), FE were reduced by 80%, from 5.4 mg/100  × g to 3 mg/100  × g (Table 1). This study demonstrated the effectiveness of IL‐modified lignin as a reactive additive in UF resin synthesis, providing both environmental and functional benefits.

Building on these findings, the research team further optimized the ionic liquid‐treated lignin–UF (LUF) resins by incorporating small amounts of isocyanate (pMDI) to enhance their properties for plywood production using the same pressing time (5 min at 1 MPa) and temperature (120 °C).^[^ ^107^ ^]^ pMDI was added at concentrations of 2%, 4%, and 6% based on resin solids, resulting in several improvements. pMDI provides highly reactive isocyanate groups that can react with both lignin and UF matrix components, forming strong urethane and urea linkages that reinforce network structure and water resistance. The inclusion of pMDI significantly increased the reactivity of the LUF resin, leading to a shorter gelation time and reduced curing temperatures. Panels bonded with the pMDI‐enhanced LUF resins exhibited 41.02% and 41.42% improvements in dry and wet shear strength, respectively, compared to those made with control LUF resins. Additionally, the pMDI‐modified resins further reduced FE by 17.14%, from 3.5 mg/100  × g to 2.9 mg/100 g, along with decreased water absorption. This work highlighted the potential of combining pMDI with IL‐modified lignin to create high‐performance, environmentally friendly wood adhesives.

Through these investigations, Younesi‐Kordkheili and his team have established a comprehensive framework for integrating modified lignins into UF resins. The key to these improvements lies in tailoring the chemical reactivity and structure of lignin to maximize its interaction with UF resin components. Their work demonstrates the versatility of lignin modification techniques, including phenolation, IL treatment, and pMDI incorporation, for improving adhesive properties, accelerating curing processes, and significantly reducing FE, making them viable solutions for sustainable wood panel production.

**Figure 6:**
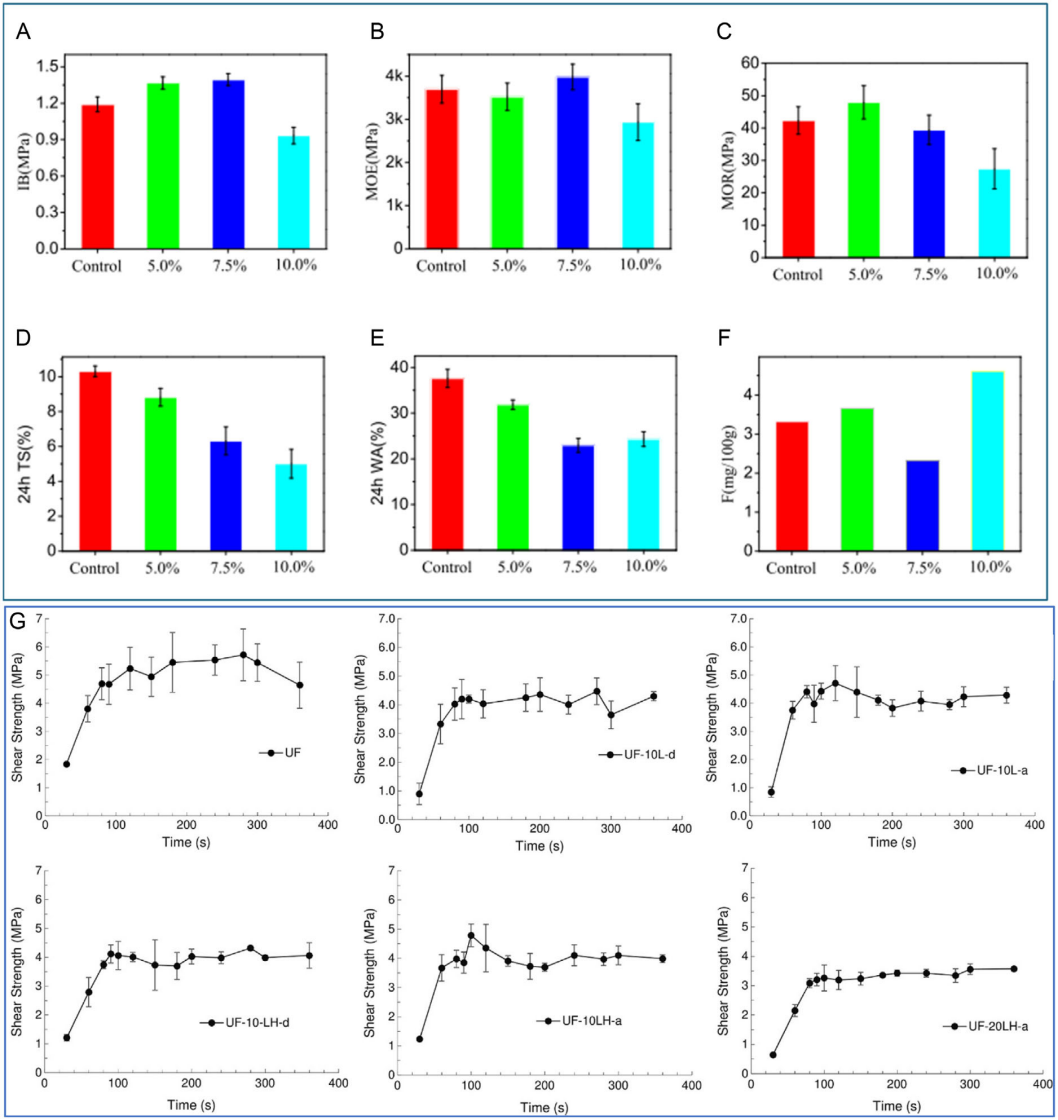
A) Internal bond strength (IB), B) modulus of elasticity (MOE), C) modulus of rupture (MOR), D) 24‐h thickness swelling (TS) rate, E) water adsorption (WA), and F) formaldehyde emission (F) of medium density fiberboard (MDF) containing various amount of hydroxymethylated lignin‐based polyacid catalyst. G) Shear strength evolution with time for the reference UF resin, UF resin with 10% of spent sulfite liquor (TSSL)/ hydroxymethylated spent sulfite liquor (TSSLH) incorporated during or postsynthesis (UF‐10 L(H)‐d and UF‐10 L(H)‐a, respectively) and UF resin with 20% of TSSLH incorporated after synthesis (UF‐20LH‐a) at a pressing temperature of 105 ºC. Reproduced with permission.^[^ ^108^ ^,^ ^109^ ^]^ Copyright 2020, MDPI; 2019, Wiley.

Recently, Gao et al.^[^ ^108^ ^]^ developed hydroxymethylated lignin‐based polyacid catalysts through a two‐step process to enhance the water resistance of UF resins. These lignin‐based catalysts promote polycondensation during UF resin formation. The hydroxymethylation introduces reactive ‐CH_2_OH groups, enabling the lignin to act both as a catalyst and cross‐linker by facilitating more efficient methylene bridge formation. The resulting UF resins exhibited reduced FE when used in plywood production (the pressing temperature was 120–125 °C hot, at 12 MPa). Moreover, lignin‐based catalysts demonstrated their potential to replace small‐molecule curing agents in UF resins, maintaining mechanical properties and reducing water absorption in medium‐density fiberboards (MDF) produced with UF resins. Panels were pressed for 200 s at 170 °C. The optimal formulation, containing 7.5% lignin‐based catalyst, led to improved mechanical and physical properties as well as enhanced FA capture, as shown in **Figure** **6**A–F.

In a related study on lignin modification, Ferreira et al.^[^ ^109^ ^]^ introduced unmodified thick spent sulfite liquor (TSSL), a by‐product of the acidic sulfite wood pulping process containing primarily lignosulfonate (58%), and hydroxymethylated TSSL (TSSLH) into a UF resin synthesis process, using two different concentrations (10% and 20%). Incorporating 10% of TSSLH after synthesis resulted in particleboards with performance comparable to those made with 90% UF resin. The pressing times and temperature were 120, 180, and 240 s at 190 °C. However, in all other tested cases, the combination of UF resin with either TSSL or TSSLH led to decreased internal mechanical properties, as shown in Figure 6G. These results indicate that the addition of TSSL or TSSLH does not improve the bonding performance of UF resin (Table 1), likely due to insufficient reactive functionalities or suboptimal integration with the UF resin matrix. However, the results indicate that a pressing temperature of 180 °C is optimal for all formulations.

It is worth noting that the hot‐pressing temperature plays a pivotal role in determining the industrial viability of lignin‐based UF adhesives. It directly influences the curing kinetics, resin penetration, and final mechanical properties of wood composites. Across the studies examined, hot‐pressing temperatures typically range from 120 °C to 190 °C, depending on the type of lignin modification and the wood product being manufactured (e.g., plywood or particleboard). For instance, plywood production with lignosulfonate or ionic liquid‐modified lignin resins was effectively conducted at relatively low temperatures (120 to 125 °C), which aligns well with energy‐saving goals in industrial settings. Conversely, particleboard panels incorporating unmodified Kraft, phenolated, or oxidized lignins required higher temperatures (190 to 220 °C) to achieve complete curing and optimal internal bond strength. These findings indicate that the hot‐pressing temperature must be carefully tailored to the resin formulation and type of lignin used, as insufficient temperature can hinder polymer crosslinking, while excessive heat may degrade lignin structure or resin performance. Therefore, integrating modified lignin into UF systems demands a holistic optimization of both chemical formulation and thermal processing parameters to ensure successful scale‐up and application.

## 4 Challenges, Environmental, and Economic Implications

### 4.1 Environmental Impacts and Life Cycle Assessment

The use of lignin as a sustainable material in UF adhesives for wood bonding has significant potential. However, it is crucial to thoroughly assess the environmental impacts associated with lignin production and utilization. This requires specific and clear metrics to ensure accurate evaluations.

LCA is the main method to quantify the environmental impacts of lignin‐based products throughout the whole value chain, from raw material extraction to end‐of‐life (EoL) disposal.^[^ ^110^ ^]^ To achieve this, environmental impacts must be allocated among the system's coproducts. The complexity of having more than one product in lignin‐based biorefineries and the fact that alternatives exist to perform this allocation, make direct comparisons challenging.^[^ ^110^, ^111^ ^]^ While LCA can determine additional impact categories such as land use, water use, and toxicity, most of the work has focused on determining global warming potential (GWP).^[^ ^112^ ^]^ A commonly used mass allocation approach has shown a GWP of ≈0.5 kg CO_2_‐eq per 1 kg of Kraft lignin^[^ ^113^ ^]^ with other studies reporting a range of 0.1 to 2.7 kg CO_2_‐eq for dry Kraft lignin.^[^ ^114^ ^]^ Organosolv lignin, derived from spruce bark (softwood), shows a GWP of 1.4–21 kg CO_2_‐eq per kg, depending on the solvent type used.^[^ ^115^ ^]^ In comparison, conventional polymers, such as polypropylene and polyethylene terephthalate (PET), have a GWPs of 1.58–1.60 kg CO_2_‐eq per kg and 55 kg CO_2_‐eq per kg, respectively.^[^ ^116^, ^117^ ^]^

In comparison, the production process of UF adhesive generates fewer greenhouse gases than PF adhesive, with GWP values of 2.04 kg CO_2_‐eq per kg for UF adhesive versus 2.88 kg CO_2_‐eq per kg for PF adhesive.^[^ ^118^ ^]^ This is due, in part, to the ability of the UF adhesive process to recycle CO_2_ for use as a raw material in urea production, along with lower energy consumption, resulting in lower GHG emissions^[^ ^118^ ^]^ The incorporation of lignin into UF adhesives offers a further opportunity to reduce the environmental impact of the adhesive. By replacing a portion of the UF resin with lignin, which has a lower GWP, it is possible to decrease the overall CO_2_ emissions associated with UF adhesive production, thus contributing to more sustainable adhesive formulations. The integration of lignin into UF adhesives not only addresses FE but also supports a reduction in the GWP of the final product, aligning with broader sustainability goals.

### 4.2 Economic and Market Analysis

**Figure 7:**
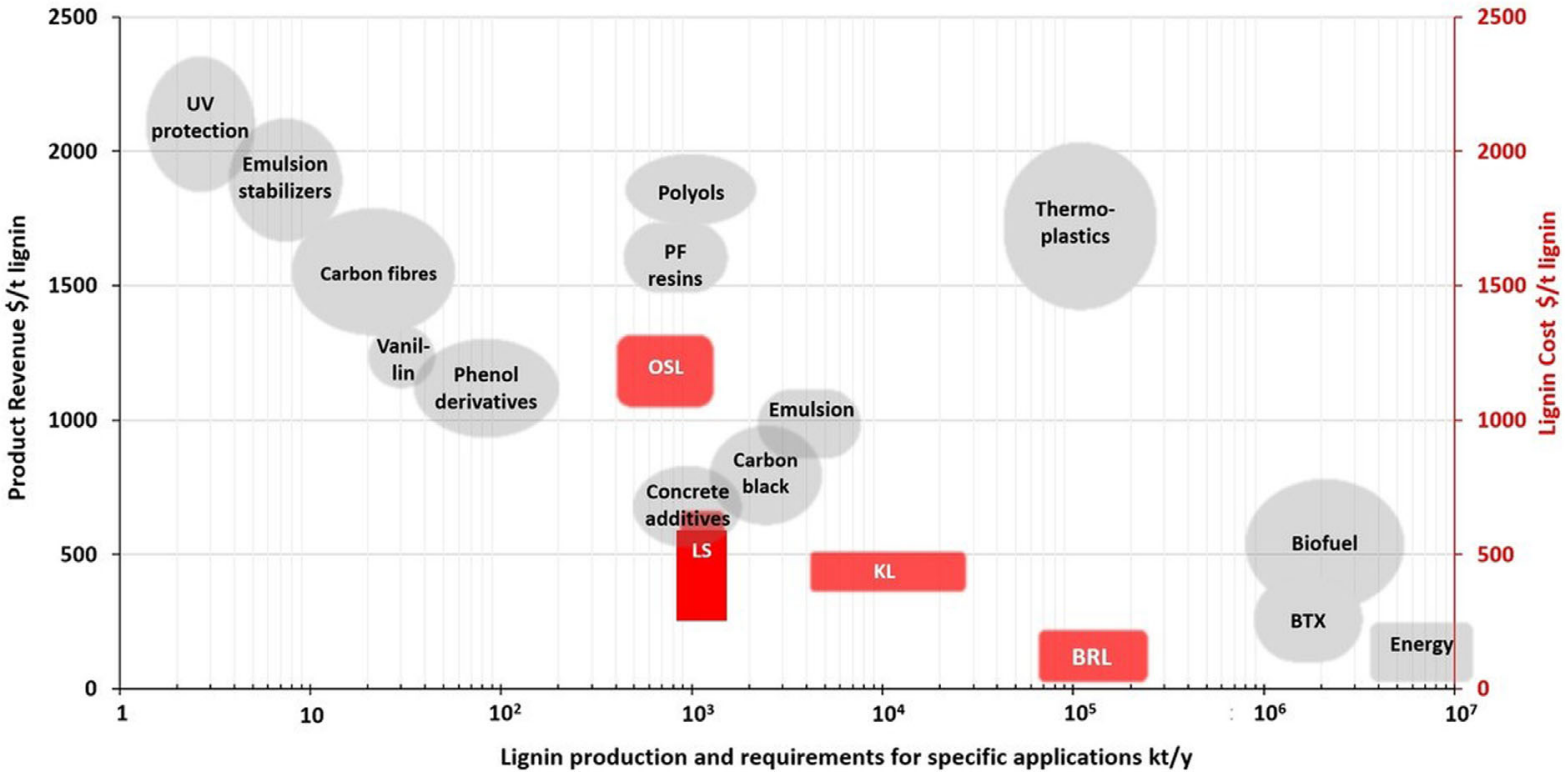
Assessment of lignin market potential using public data. Abbreviations: OSL–organosolv lignins, LS–lignosulfonates, KL–Kraft lignins, BRL (crude) biorefinery lignins. Reproduced with permission.^[^ ^111^ ^]^ Copyright 2021, Royal Society of Chemistry.

The economic strategies for valorizing different types of lignin across various applications are shown in a plot that illustrates estimated lignin production costs and volumes compared to potential application volumes and revenues (**Figure** **7**).

Figure 7 highlights the general tendencies in lignin costs and production volumes, emphasizing that these values are approximations due to the nascent market. Considering both material costs and the additional expenses associated with lignin recovery versus incineration is essential. While crude biorefinery lignins (BRLs) are not pure lignin, they frequently contain significant residual sugar content and other impurities from biomass processing. They often have low costs, these can sometimes be negative when incineration costs surpass lignin's energy value.^[^ ^119^ ^]^ BRLs are advantageous over other lignin types, especially when used without further processing, making various applications economically feasible.^[^ ^120^, ^121^ ^]^

The cost of lignin extraction varies depending on the applied process. Kraft lignin recovered from black liquor through acid precipitation generally costs between USD 200–400 per metric ton, while organosolv lignin may cost between USD 600–1200 per metric ton due to the higher cost of solvents and purification steps.^[^ ^122^, ^123^ ^]^ Additionally, a techno–economic assessment of industrial‐scale lignin modification for epoxy resin applications has shown that the production cost of epoxidized Kraft lignin can range between USD 1100–1300 per ton, depending on plant scale, energy use, and reagent consumption.^[^ ^124^ ^]^ This is in line with other reports showing that chemical modifications such as hydroxymethylation introduce an additional processing cost of USD 100–300 per ton, depending on reaction conditions and batch size.^[^ ^80^, ^124^ ^]^

In comparison, the production cost of conventional UF resin is estimated at USD 600–900 per ton.^[^ ^125^, ^126^ ^]^ Partial substitution of UF resin with lignin, typically 10%–20%, has been shown to reduce resin cost by ≈10%–15%, particularly when using crude Kraft lignin without purification.^[^ ^127^ ^]^ Although chemical modification adds cost, the overall economics remain favorable when lignin is sourced from existing pulp and paper operations. In some scenarios, lignin‐based UF adhesives have demonstrated equivalent or lower unit production costs compared to traditional UF resins, particularly when environmental benefits (e.g., FE reductions) are considered.^[^ ^124^ ^]^

High‐value, small‐market applications, such as biomedical uses, are attractive for niche producers but not for bulk lignin producers. Conversely, high‐volume, low‐price applications (e.g., fuels) benefit customers seeking sustainable materials but are less profitable for producers. The optimal market for lignin producers lies in high‐value applications that can replace traditional raw materials, such as phenol, polyols, Bisphenol A, and polyacrylonitrile (PAN), as well as major product categories like thermosets and thermoplastics. As technology and markets evolve, there is potential for lignin to be utilized in producing phenolic resins, polyurethane, epoxy resins, composites, and other additives.^[^ ^122^ ^]^

Thermoplastics are particularly promising due to their large market and good value. With high production costs, Organosolv lignins (OSLs) should target markets where their superior performance justifies the price. Polymeric lignins can be cost‐effective for nanocomposites, but they face challenges due to their heterogeneous structure and low purity, necessitating careful volume management to enhance benefits.

In summary, despite being a renewable biopolymer, few lignin applications have been commercially successful, aside from its use for energy applications. However, as a complex phenolic macromolecule, lignin offers many opportunities for creating advanced sustainable materials. These opportunities depend on the extraction methods established under biorefinery concepts to ensure process sustainability.

## 5 Conclusions

With their distinct chemical characteristics and encouraging reactivity, lignins have become very attractive biomaterials with various possible uses in wood adhesive formulations. This review has offered a thorough exploration of recent research, illuminating the multifaceted role of lignin partially substituting UF adhesives or urea during UF synthesis process. Lignin has proven to be highly versatile and valuable in the wood industry, whether used as a partial replacement or incorporated during resin synthesis. The significant advancements in lignin‐based UF adhesives over the past decade highlight the increasing interest and potential in this area.

This review has emphasized the importance of utilizing lignin, a natural adhesive component in wood, to reduce the amount of synthetic UF resin in wood panels. Research indicates that lignosulfonates, soda lignin, and Kraft lignin are particularly suitable for this application due to their chemical properties and availability. Studies have shown that lignin can replace up to 20%–30% of UF resin, with a solid content between 50%–60%, without significantly compromising panel performance, in some cases even improving mechanical properties and reducing FE.

However, gaps in the literature remain, particularly regarding the long‐term performance, scalability, and economic viability of lignin‐UF hybrid systems. Future research should focus on optimizing lignin modification techniques, improving lignin‐UF compatibility, and conducting comprehensive life cycle assessments. Additionally, pilot‐scale studies and industry collaborations are crucial to encourage panel producers to adopt lignin on a commercial scale.

Beyond its technical advantages, lignin represents a sustainable alternative, derived from renewable, eco‐friendly materials, aligning with green chemistry and environmental goals. This review underscores lignin's potential in advancing wood adhesive technology while highlighting the need for continued research to address existing challenges and promote widespread industrial adoption.

## Conflict of Interest

The authors declare no conflict of interest.
